# Transcriptomics and Proteomics Analysis of the Liver of *RAD52* Knockout Mice

**DOI:** 10.3390/ijms26010339

**Published:** 2025-01-02

**Authors:** Yingjie Song, Lan Yang, Yao Han, Wenjun Li, Tong Wei, Yamin Gao, Qiang Hu, Hao Li, Yansong Sun

**Affiliations:** State Key Laboratory of Pathogen and Biosecurity, Academy of Military Medical Sciences, Beijing 100071, China; ylsong72@163.com (Y.S.); poplarorchid@163.com (L.Y.); hanyaohyhy@163.com (Y.H.); liwenjun181@mails.ucas.ac.cn (W.L.); jw03421kt@163.com (T.W.); gymin09@163.com (Y.G.); hq2211295426@163.com (Q.H.)

**Keywords:** RAD52, oxidative phosphorylation, neurodegenerative diseases, nucleotide excision repair, transcriptome, data-independent acquisition

## Abstract

RAD52 plays crucial roles in several aspects of mammalian cells, including DNA double-strand breaks repair, viral infection, cancer development, and antibody class switching. To comprehensively elucidate the role of RAD52 in maintaining genome stability and uncover additional functions of RAD52 in mammals, we performed the transcriptomics and proteomics analysis of the liver of *RAD52* knockout mice. Transcriptomics analysis reveals overexpression of mitochondrial genes in the liver of *RAD52* knockout (RAD52KO) mice. Proteomics analysis of RAD52KO mice shows that damage recognition proteins Cul4b and Rad23a in the process of nucleotide excision repair pathway are overexpressed. Furthermore, gene ontology and KEGG enrichment analysis (accessed on 20 November 2024) from integrated omics shows that differentially expressed genes are significantly enriched in pathways related to mitochondrial oxidative phosphorylation and nucleotide metabolism in the liver of RAD52KO mice. In addition, mRNA and protein levels of Bhmt1b are elevated in the liver of RAD52KO mice. Taken together, this study provides valuable insights into the function and mechanism of RAD52.

## 1. Introduction

RAD52 was first identified along with homologous recombination repair (HRR) proteins in a screen for DNA repair-deficient *Saccharomyces cerevisiae* mutants exposed to ionizing radiation [1]. In yeast, RAD52 is considered to be the most important DNA repair gene, and its activity is required for almost all fungal repair and recombination reactions [2,3,4]. *RAD52* mutants in *Saccharomyces cerevisiae* and *Naumovozyma castellii* exhibit severe defects in double-strand break (DSB) repair [5,6]. Subsequently, RAD52 ortholog was identified in eukaryotes. While previous studies demonstrated that RAD52 is dispensable for eukaryotic HRR by *RAD52* deletion in terms of phenotype [7,8], the impact of mammalian RAD52 on cellular metabolism remained unknown. However, recent findings indicate that RAD52 participates in HRR [9,10], single-strand annealing [11,12], break-induced replication [13], the occurrence and development of cancer [14,15,16,17], viral infection, and antibody class switching [18,19], suggesting diverse and significant roles for RAD52 in mammalian cells.

Most of *RAD52* gene functions were found in the process of exploring the mechanism of certain phenomena, and RAD52 was screened to play important roles in the process. For example, CRISPR-Cas9 knockout screens revealed a synthetic lethal interaction between RAD52 inactivation and BRCA1/2 mutations in tumors, suggesting novel therapeutic target [13]. However, a comprehensive analysis of RAD52 function from a gene ontology perspective remains lacking.

Transcriptomics and proteomics analysis provides comprehensive information on gene expression and protein abundance, enabling the elucidation of RAD52’s functions and regulatory networks. Given the liver’ central role in metabolism and its susceptibility to DNA damage accumulation, it serves as a suitable model for investigating the functions of the DNA repair gene *RAD52* [20,21,22]. In this study, we performed the transcriptomics and proteomics analysis of the liver of *RAD52* knockout mice to investigate *RAD52* gene function. Transcriptomics analysis revealed increased mRNA expression of mitochondrial genes in RAD52KO mice. Proteomic analysis showed overexpression of the nucleotide excision repair pathway proteins Cul4b and Rad23a. Additionally, integrated bioinformatic analysis suggested that the expression of Bhmt1b mRNA and protein increases in the liver of RAD52KO mice. The transcriptomics and proteomics analysis contributes to a better understanding of RAD52’s role in maintaining genome stability and provides insights into novel RAD52 functions in mammals.

## 2. Results

### 2.1. RAD52 Knockout Causes Differential Gene Expression in the Transcriptome of the Liver

**Figure 1:**
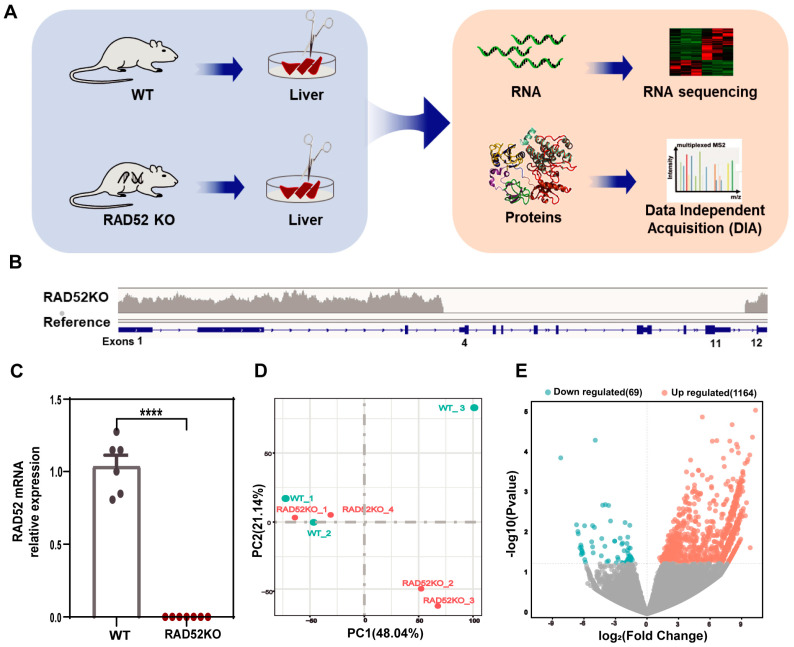
*RAD52* knockout causes differential gene expression in the transcriptome of the liver. (**A**) The flow diagram in this study. RAD52KO represents *RAD52*−/− knockout mice; WT represents wild-type mice. (**B**) The results of whole-genome sequencing showed the *RAD52* gene on the *RAD52*−/− knockout mice and the mouse reference genome (GRCm38). Blue rectangles represent exons; the grey bar represents the intensity of the sequencing signal. (**C**) The mRNA levels of RAD52 were performed between the RAD52KO group (*n* = 6) and WT group (*n* = 6) by RT-qPCR. The data are shown as mean ± SEM. *p*-values were calculated using an unpaired *t* test; **** *p* < 0.0001. (**D**) The results of PCA for *RAD52*−/− knockout mice (*n* = 4) and wild-type mice (*n* = 3) in RNA-Seq. PC represents principal component. (**E**) Volcano plots show the differentially expressed genes between the RAD52KO and the WT group.

To explore the functions of the *RAD52* gene in mammals, we used *RAD52*−/− knockout mice to investigate the effects of RAD52 deletion. As illustrated in Figure 1A, *RAD52*−/− knockout mice and wild-type mice were executed, and livers were ground into single-cell suspensions. Total RNA and proteins were then extracted for RNA and proteomic sequencing, respectively. We analyzed the transcriptomics data, followed by the proteomics data, and finally integrative omics to explore the potential roles of the *RAD52* gene. To confirm homozygous knockout of the *RAD52* gene, we performed whole genome sequencing. The results showed that the region of the *RAD52* gene between exon 4 and exon 11 had no read coverage (Figure 1B). To further verify the expression of the *RAD52* gene, we performed quantitative real-time PCR (RT-qPCR) experiments. The results of RT-qPCR demonstrated that the RAD52 mRNA is unable to be expressed normally (Figure 1C). Therefore, *RAD52* knockout mice have been successfully constructed.

By principal component analysis (PCA) of RNA-Seq, the results showed that 48.04% (PC1) and 21.04% (PC2) accounted for the total variance. The WT-3 sample was distant from the other wild-type samples on PC1 and PC2, and excluded from subsequent analysis (Figure 1D). RAD52KO-1 and RAD52KO-4 were close to the wild-type, while the other two were distant. The transcriptomics analysis identified 23,030 genes, with 1233 differentially expressed genes (DEGs). Specifically, 1164 genes were found to be upregulated, and 69 genes were downregulated. DEGs are mainly upregulated expression genes (Figure 1E). The results showed that *RAD52* knockout caused differential gene expression in the transcriptomics analysis.

### 2.2. Mitochondrial Genes Were Overexpressed in the Transcriptomics Analysis of the Liver of RAD52KO Mice

**Figure 2:**
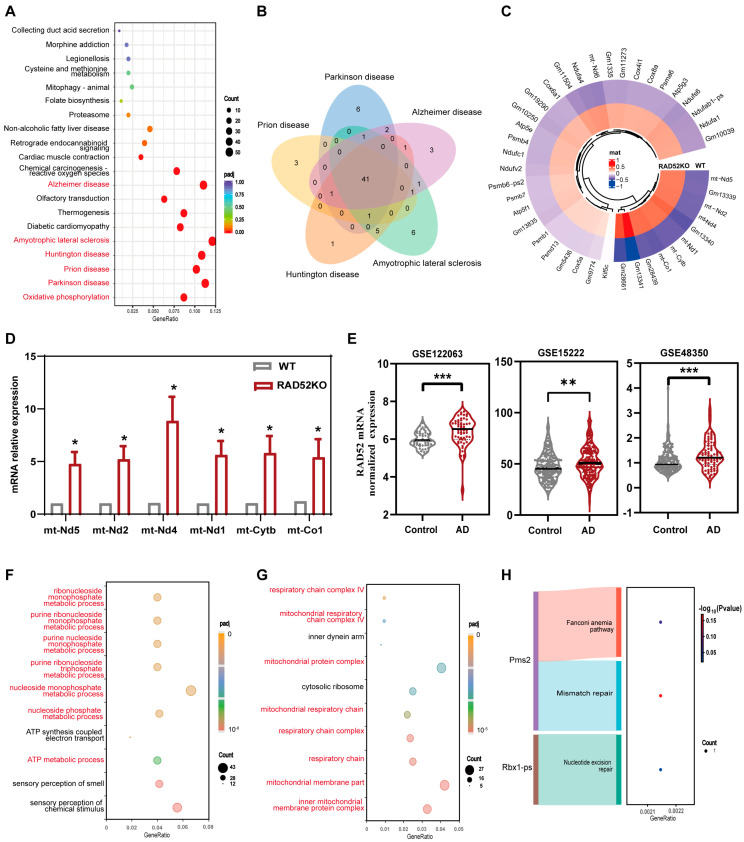
Mitochondrial genes were overexpressed in the transcriptomics analysis of the liver of RAD52KO mice. (**A**) Bubble diagram of the top 20 KEGG pathways. padj represents the adjusted *P*-value. GeneRatio represents the ratio of target genes enriched in the pathway among genes annotated in the pathway. (**B**) Venn analysis shows the numbers of shared genes in the neurodegenerative diseases. (**C**) Cluster analysis of shared genes in the neurodegenerative diseases. Mat represents matrix. (**D**) The mRNA levels of the mitochondrial genes were performed between the RAD52KO group (*n* = 6) and WT group (*n* = 8) by RT-qPCR. The data are shown as mean ± SEM. *p*-values were calculated using an unpaired *t* test; * *p* < 0.05. (**E**) The normalized expression of RAD52 mRNA between the AD group and Control group using sequencing data of Gene Expression Omnibus (GEO) related to neurodegenerative diseases. AD represents the AD group sampled from people with Alzheimer’s disease; Control represents the Control group sampled from people without Alzheimer’s disease. *p*-values were calculated using an unpaired *t* test; ** *p* < 0.01, *** *p* < 0.001. (**F**) The top 10 pathways of GO term for biological process. (**G**) The top 10 pathways of GO term for cell component. (**H**) The results of KEGG enrichment on the DEGs related to DNA repair pathways.

To elucidate the functions of these DEGs, we performed KEGG pathway enrichment and GO term enrichment analysis. The results of KEGG enrichment analysis showed that the DEGs were primarily enriched in the pathways of oxidative phosphorylation and neurodegenerative diseases (Figure 2A). Those neurodegenerative diseases included Parkinson’s disease, prion disease, Huntington’s disease, amyotrophic lateral sclerosis and Alzheimer’s disease. Subsequently, to explore whether RAD52 affects shared genes in these neurodegenerative diseases, Venn analysis was conducted on the DEGs from five neurodegenerative diseases to identify shared differential genes. The results showed that there were 41 shared DEGs in the neurodegenerative diseases, of which 37 of the 41 genes are oxidative phosphorylation pathway (Figure 2B). Cluster analysis of those genes showed that 41 genes were overexpressed in *RAD52* knockout mice compared to wild-type mice. Among these, several mitochondrial genes (mtDNA), including *mt*-*Nd5*, *mt*-*Nd2*, *mt*-*Nd4*, *mt*-*Nd1*, *mt*-*Cytb*, and *mt*-*Co1*, exhibited significant expression (Figure 2C).

Previous studies have demonstrated that mitochondrial genes play important roles in the process of neurodegenerative diseases [23,24,25,26,27]. We detected mRNA levels of the significant mitochondrial genes using RT-qPCR. The results confirmed that the mRNA levels of those genes were overexpressed in the *RAD52* knockout group compared to the WT group (Figure 2D), which was consistent with the transcriptomics results. In addition, to further explore whether RAD52 is associated with neurodegenerative disease, we analyzed the normalized expression of RAD52 mRNA between the AD group and the control group using the data from Gene Expression Omnibus (GEO) (accessed on 5 November 2024) related to neurodegenerative diseases. According to the results of GSE122063 [28], GSE15222 [29] and GSE48350 [30] analysis, RAD52 mRNA was overexpressed in the Alzheimer’s disease group compared to Control group sampled from people without Alzheimer’s disease (Figure 2E).

To further verify the mitochondrial function affected by RAD52, GO term enrichment analysis was performed on the DEGs identified from the transcriptomics analysis, focusing on enrichment in the biological process and cell component. The biological process (BP) analysis showed that the DEGs are significantly involved in sensory perception of chemical stimuli, sensory perception of smell, and ATP metabolic process, with the first two being related to the nervous system. The majority of the enriched processes were associated with nucleotide metabolism, including the nucleoside monophosphate metabolic process and the purine nucleoside monophosphate metabolic process (Figure 2F). Additionally, the cell component (CC) analysis showed that the DEGs are significantly involved in the mitochondrial membrane part and the respiratory chain complex (Figure 2G).

Given the pivotal functions of RAD52 in DNA repair pathways, we performed KEGG enrichment analysis on the DEGs related to DNA repair pathways. The results of enrichment analysis showed that those DEGs were primarily enriched in the Fanconi anemia pathway, mismatch repair, and nucleotide excision repair (Figure 2H). The differentially expressed genes included *Pms2* and *Rbx1*-*ps*.

### 2.3. RAD52 Knockout Causes Differential Protein Expression and Affects the Expression of Nucleotide Metabolism in the Proteome of the Liver

**Figure 3:**
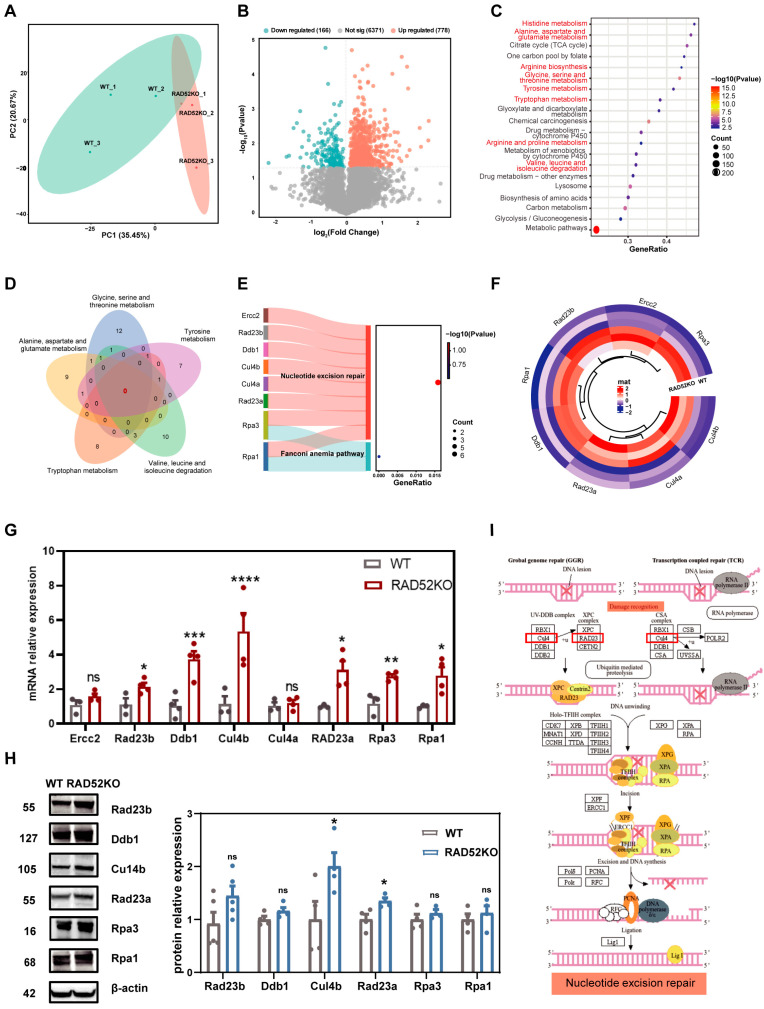
*RAD52* knockout causes differential protein expression and affects pathway related to nucleotide metabolism and nucleotide excision repair in the proteome of the liver. (**A**) The results of PCA for RAD52−/− knockout mice (*n* = 3) and wild-type mice (*n* = 3) in proteomic sequencing. (**B**) Volcano plots show the DEPs between the RAD52KO and the WT group in DIA analysis. (**C**) Bubble diagram of the top 20 KEGG pathways. (**D**) Venn analysis showed the numbers of shared proteins in the metabolic pathways. (**E**) The results of KEGG enrichment on the differentially expressed proteins related to DNA repair pathways. (**F**) Cluster analysis of the differentially expressed proteins related to DNA repair pathways. Mat represents matrix. (**G**) The mRNA levels of the proteins were performed between the RAD52KO group and WT group by RT-qPCR. The data are shown as mean ± SEM. *p*-values were calculated using an unpaired *t* test; ns *p* > 0.05, * *p* < 0.05, ** *p* < 0.01, *** *p* < 0.001, **** *p* < 0.0001. (**H**) The expression of the proteins was tested between the RAD52KO group and WT group by Western blot. Beta actin was used as an endogenous gene. The data are shown as mean ± SEM. *p*-values were calculated using an unpaired *t* test; ns *p* > 0.05, * *p* < 0.05. (**I**) Overexpression of proteins in nucleotide excision repair pathway were shown using the KEGG pathway database. Red rectangular box represents overexpression of proteins.

By principal component analysis of proteomics analysis, the results showed that 35.45% (PC1) and 20.67% (PC2) accounted for the total variance. The *RAD52* knockout group and WT group showed good reproducibility, with high variability between them (Figure 3A). The proteomics analysis identified 7315 proteins, with 944 differentially expressed proteins (DEPs). Specifically, 778 proteins of these proteins were overexpressed, while 166 proteins of these were downregulated (Figure 3B). DEPs predominantly revealed overexpressed proteins, which were consistent with the results of transcriptomics analysis. However, the fold changes observed in the transcriptomics analysis predominantly ranged from 1 to 9 (Figure 1C), whereas the proteomics analysis revealed fold changes primarily between 0 and 3 (Figure 3B), showing that the fold changes in the proteomic data were significantly lower than those in the transcriptomic data.

To interpret the functions of these DEPs in the proteome, we performed KEGG pathway enrichment analysis. The top 20 results of KEGG enrichment analysis showed that the DEPs were primarily enriched in metabolic pathways (Figure 3C). The top three pathways of metabolic pathways, ranked by *p*-value, included glycine, serine, and threonine metabolism; alanine, aspartate, and glutamate metabolism; and tryptophan metabolism. Subsequently, to explore whether RAD52 affects shared genes in these metabolic pathways, Venn analysis was conducted on the DEPs from the top five metabolic pathways to identify shared differential proteins. The results showed that there was no shared differentially expressed protein among the metabolic pathways in the RAD52KO mice (Figure 3D).

### 2.4. The Expression Levels of Cul4b and Rad23a Protein Increase in the Proteome of RAD52KO Mice

Given the important roles of RAD52 in DNA repair pathways, we performed KEGG enrichment analysis on the DEPs related to DNA repair pathways. The results showed those proteins were enriched in the nucleotide excision repair and Fanconi anemia pathway (Figure 3E). Both transcriptomics and proteomics sequencing were enriched in these pathways. The differentially expressed proteins included Rpa1, Rpa3, Rad23a, Cul4a, Cul4b, Ddb1, Rad23b, and Ercc2. Subsequently, clustering analysis of the differentially expressed proteins showed that the protein expression levels of the DNA repair pathway increased (Figure 3F).

Furthermore, RT-qPCR confirmed that the mRNA levels of Rpa1, Rpa3, Rad23a, Cul4b, Ddb1, and Rad23b were overexpressed in the *RAD52* knockout group compared to the WT group (Figure 3G). In addition, the results of Western blot showed that Cul4b and Rad23a were overexpressed in the *RAD52* knockout group (Figure 3H). The roles of Cul4b and Rad23a in nucleotide excision repair pathway were shown using the KEGG pathway database (Figure 3I). Cul4b and Rad23a proteins are involved in damage recognition, which is the initial step of the nucleotide excision repair pathway. Proteomic analysis of *RAD52* knockout mice suggested that the expression of Cul4b and Rad23a in the nucleotide excision repair increased in the liver RAD52KO mice.

### 2.5. Bhmt1b Is Overexpressed in the Transcriptomics and Proteomics Analysis of RAD52 Knockout Mice

**Figure 4:**
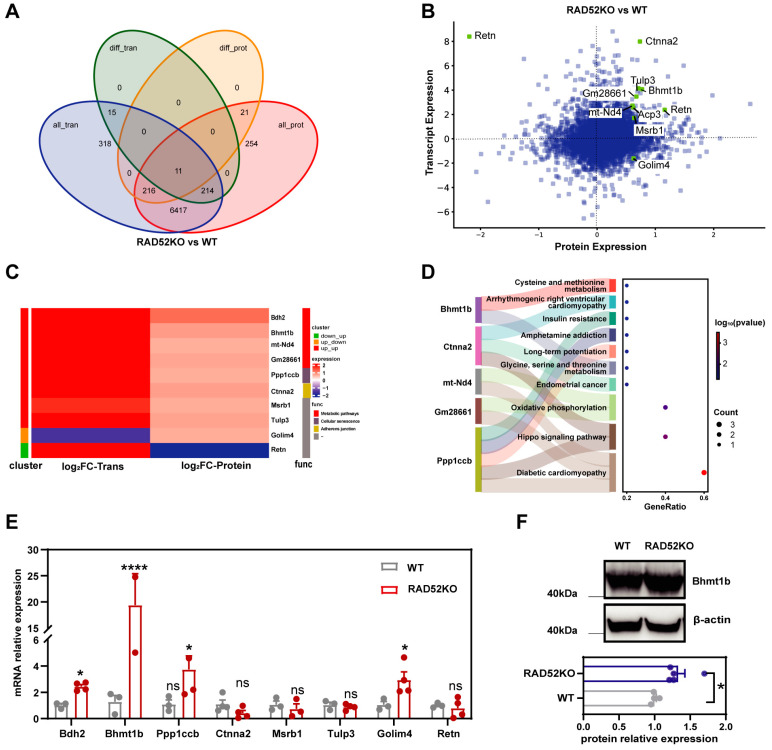
Bhmt1b is overexpressed in the transcriptomics and proteomics analysis of *RAD52* knockout mice. (**A**) Venn analysis showed the numbers of shared genes in the transcriptomics and proteomics sequencing. (**B**) The distribution of fold changes in transcriptomic and proteomic data. (**C**) Cluster analysis of shared DEGs. Func represents function. (**D**) The results of KEGG enrichment on the shared DEGs. (**E**) The mRNA levels of the shared genes were performed between the RAD52KO group (*n* = 4) and the WT group (*n* = 4) by RT-qPCR. The data are shown as mean ± SEM. *p*-values were calculated using an unpaired *t* test; ns *p* > 0.05, * *p* < 0.05, **** *p* < 0.0001. (**F**) The relative expression of Bhmt1b protein by Western blot. Beta actin was used as an endogenous gene. The data are shown as mean ± SEM. *p*-values were calculated using an unpaired *t* test; * *p* < 0.05.

To comprehensively explore the function of RAD52, we performed integrated analysis both in the transcriptomics and proteomics analyses following *RAD52*−/− knockout. Initially, we performed Venn analysis on all genes and differentially expressed genes identified by transcriptomic and proteomics analysis. The results showed that 11 genes were identified in integrated omics analysis, including *Bdh2*, *Bhmt1b*, *mt*-*Nd4*, *Gm28661*, *Ppp1ccb*, *Ctnna2*, *Msrb1*, *Tulp3*, *Golim4*, and *Retn* (Figure 4A,B). The distribution of fold changes in transcriptomics and proteomics data showed that eight genes were consistently upregulated. Notably, *Retn* gene was upregulated in the transcriptomics analysis with a fold change of 8, while it was downregulated in the proteomics analysis, showing a significant fold change of 2.2 relative to other differentially expressed proteins in the proteomic data. The *Golim4* gene was downregulated in the transcriptomics analysis with a fold change of 1.6, while it exhibited upregulation in the proteomics analysis with a fold change of 0.6 (Figure 4C).

Subsequently, KEGG enrichment analysis of those genes showed that only five genes could be annotated to KEGG pathways (Figure 4D). These genes included *Bhmt1b*, *Bdh2*, *mt*-*Nd4*, and *Gm28661*, all of which are involved in metabolic pathways. Notably, *mt*-*Nd4* and *Gm28661* are genes associated with the mitochondrial oxidative phosphorylation process.

To confirm the expression of eight genes at the mRNA levels, data were validated using RT-qPCR (Figure 4E). The results indicated that the *RAD52* knockout group exhibited upregulation of Bhmt1b, Bdh2, Ppp1ccb, and Golim4 compared to wild-type mice. The mRNA level of Golim4 in the *RAD52* knockout group was three times that of the control group, which was inconsistent with transcriptomics analysis. Notably, the mRNA expression level of Bhmt1b was found to be 19 times higher than that of the control group. Bhmt1b plays crucial roles in the remethylation of homocysteine to methionine, which is an essential step in the one-carbon metabolism pathway. We further verified the expression of Bhmt1b protein by Western blot. The results showed that Bhmt1b protein was overexpressed in the RAD52KO group compared to the WT group (Figure 4F). Therefore, the findings suggested that there might be a relationship between RAD52 and Bhmt1b.

## 3. Discussion

RAD52 plays important roles in DNA repair, cancer development, and antibody class switching. To comprehensively explore the function of RAD52, we performed transcriptomic and proteomics analysis of the liver of *RAD52* knockout mice. Our findings show that mitochondrial genes are overexpressed in the liver of RAD52KO mice. In the proteomics analysis of the liver of RAD52KO mice, the expression of Cul4b and Rad23a proteins increases in the damage recognition process of nucleotide excision repair pathway. In addition, Bhmt1b mRNA and protein are overexpressed in the liver of RAD52KO mice.

In the PCA results of the transcriptome data, two samples from the RAD52KO group deviated from the other two replicates in that group. By analyzing the differential genes between replicates, the results show that differential genes are mainly pseudogenes and do not affect the conclusions presented in the study. As for changes in the proteomic data, they are significantly different from those in the transcriptomic data; potential explanations for this discrepancy include post-transcriptional regulation, translational efficiency, technical variability, bioinformatics and data normalization, and regulatory feedback mechanisms.

Previous studies have identified RAD52 as an important protein in the repair of mitochondrial DNA damage [31,32]. Mitochondrial dysfunction has been recognized as a critical factor in neurodegenerative mechanisms [25]. However, the potential connection between RAD52 and neurodegenerative diseases remains unknown. Interestingly, this study found that mitochondrial genes of the oxidative phosphorylation process are overexpressed in RAD52KO mice. We hypothesize that *RAD52* deletion might lead to mitochondrial dysfunction, subsequently contributing to the development of neurodegenerative diseases. Interestingly, analysis of sequencing data from the GEO database related to neurodegenerative diseases reveals a potential link between RAD52 and neurodegenerative diseases. This highlights the significance of RAD52 in maintaining neuronal integrity and its potential role in the progression of neurodegenerative diseases.

In addition, RAD52 plays crucial roles in DNA repair pathways, including HRR, transcription-associated HRR, and single-strand annealing. We found that Rad23a and Cul4b proteins of the damage recognition process in the nucleotide excision repair pathway are overexpressed in RAD52KO mice. We hypothesize that the expression of RAD52 might trigger or regulate the initiation of nucleotide excision repair, thereby affecting the activation state and function of the entire pathway. When the organism is faced with bulky DNA lesions resulting from both environmental exposures and endogenous metabolic processes, RAD52 could potentially enhance NER repair and HRR capacity to maintain genome stability by regulating RAD52 expression. The assumption needs to be further validated.

Recently, the connection between metabolic dysfunction and inflammation has garnered attention, particularly regarding its implications for neuroinflammation and neurodegenerative diseases [33]. This connection is particularly relevant given the liver’s central functions in metabolic regulation and its vulnerability to various metabolic disorders. *RAD52*, which is a key gene involved in DNA repair, may also play pivotal roles in the liver’s response to metabolic stress. When metabolic dysfunction occurs, the liver undergoes a stress state, resulting in cellular damage and activation of inflammatory pathways. This systemic inflammatory reaction has a profound impact on the central nervous system. Under these conditions, RAD52 might be upregulated and respond to DNA damage to maintain genomic stability, which is essential for proper liver and brain functions.

Previous studies have identified a compensatory relationship between RAD52 and BRCA2 [4,34,35,36,37]. The absence of RAD52 is lethal to cells with BRCA2 deficiencies [4,35,36]. In this study, we found overexpression of Bhmt1b mRNA and protein in the liver of RAD52KO mice. The results suggest that RAD52 and Bhmt1b might have a potential compensatory relationship. Bhmt1b primarily participates in choline metabolism, with no identified intermediaries linking its function to that of *RAD52* gene. We proposed the hypothesis that RAD52 and Bhmt1b may compensate for each other in cellular stress responses. This compensatory relationship could have important implications for cancer therapies. When RAD52 is overexpressed in certain tumors, inhibition of RAD52 may enhance the therapeutic sensitivity of tumor cells. At the same time, the activity of Bhmt1b could serve as a biomarker for assessing treatment efficacy. The hypothesis needs to be further verified. In summary, these findings systematically explore the roles of RDA52 and provide a reference for the discovery of novel functions of RAD52.

## 4. Materials and Method

### 4.1. RAD52−/− Knockout Mice

*RAD52* heterozygous mutant mice were constructed by CRISPR/Cas9 gene-editing technology targeting exon4-exon11 of the *RAD52* gene by GemPharmatech Co., Ltd., Nanjing, China. The targeting vector was electroporated into C57BL/6JGpt mouse zygotes, and germline transmission was achieved in two independent founder lines. RAD52−/− mice were produced by heterozygous–heterozygous mating at Beijing Vital River Laboratory Animal Technology Co., Ltd., Beijing, China. Mouse genotypes were identified by PCR method and primers were shown in Table 1. Genomic DNA were isolated from the tails, mouse genotypes were identified by PCR method, and primers are shown in Table 1. The amplification results of PCR-1 were 10178bp (wild-type) and 347bp (RAD52−/−) products, and the amplification results of PCR-2 were 305bp (wild-type) and 0bp (*RAD52* knockout) products. Twenty-week-old male *RAD52* knockout and wild-type mice were sacrificed, and liver samples were collected for subsequent sequencing.

### 4.2. Whole-Genome Sequencing

Mouse DNA from liver cells was used as input material for the DNA library preparations. Library quality was assessed on the Agilent 5400 system (Agilent, Santa Clara, CA, USA) and quantified with higher than 1.5 nM. The qualified libraries were pooled and sequenced on Illumina platforms with PE150 strategy by Novogene Co., Ltd., Beijing, China. Paired reads were discarded if more than 10% of the bases were uncertain in each read and if the proportion of low-quality (Phred quality < 5) bases was over 50% in each read. Valid sequencing data were mapped to the reference genome (GRCm38) by Burrows–Wheeler Aligner (BWA) software (v0.7.17) and dislodged duplication by SAMtools to obtain the mapping results stored in BAM format. Finally, the results of bam files were visualized with the Integrative Genomics Viewer (IGV).

### 4.3. Transcriptomic Sequencing

Total RNA from mouse liver was extracted using Tissue RNA Extraction Kit (Tiangen, Beijing, China), according to the manufacturer’s instructions. mRNA was purified using poly-T oligo-attached magnetic beads, and mRNA libraries with effective concentrations higher than 1.5 nM were subsequently selected. The libraries were sequenced on an Illumina Novaseq platform by Novogene Co., Ltd., Beijing, China. FeatureCounts (v1.5.0) was used to count the read numbers mapped to each gene. Sequencing data were filtered to remove low-quality reads with Qphred <= 5 bases accounting for more than 50% of the total read length. The FPKM of each gene was calculated based on the length of the gene and read count mapped to that gene. Genes with an adjusted *p*-value ≤ 0.05 and |log_2_FC| > 0 were assigned as differentially expressed genes. Volcano plot analysis, cluster analysis, GO term, and KEGG enrichment analysis of differentially expressed genes were performed using the NovoMagic tools (https://magic.novogene.com/ (accessed on 20 November 2024)). Volcano plot and cluster heatmap results were visualized by http://www.bioinformatics.com.cn (accessed on 4 November 2024), an online platform for data analysis and visualization. 

### 4.4. Data-Independent Acquisition (DIA)

Mouse liver was ground into single-cell suspensions, and total protein was extracted for mass spectrometric analysis. Proteomic sequencing was performed by Novogene Co., Ltd. The cleared protein samples were analyzed using a mass spectrometer (Thermo Orbitrap Astral) to generate mass spectrometric data. DIA-NN software (v1.9.2) was employed to analyze the raw data. Analysis parameters were PG. Q.Value < 0.05, Q.Value < 0.01, and Global.PG.Q.Vlaue < 0.01, and only credible spectroscopic peptides and proteins were retained. The results were further filtered by removing spectral peptides with a confidence level of less than 1% and peptides and proteins with an FDR greater than 1%. Proteins with a *p*-value ≤ 0.05 and |log_2_FC| > 0 were assigned as differentially expressed proteins. The results of volcano plot analysis, cluster analysis, GO term, and KEGG enrichment analysis were visualized on the website, which was consistent with that in the transcriptomics analysis. 

### 4.5. Quantitative Real-Time PCR (RT-qPCR)

Mouse liver was ground at -10 °C by Freeze Grinder (Jingxin, Shanghai, China), and total RNA was extracted using Tissue RNA Extraction Kit (Tiangen, Beijing, China). Two-step RT-qPCR was performed on a CFX96 Real-Time Analyzer (Bio-Rad, Hercules, CA, USA) using reverse transcription reagents (Accurate Biology, Hunan, China) and SYBR green premixed reagents (Accurate Biology, Hunan, China). The primers are listed in Table 2, and *β*-*actin* was used as the reference gene. The data were analyzed using the 2^−∆∆Ct^ method [38]. 

### 4.6. Western Blot

Mouse liver was ground at −10 °C with a freeze-grinding instrument (Jingxin, Shanghai, China). Total proteins of liver cells were lysed by Pierce™ IP lysate buffer (Thermo, 87787) with 1% protease inhibitor cocktail (Thermo Fisher Scientific, Waltham, MA, USA). After being centrifuged at 14000× g for 10 min, the supernatant was filtered using a 0.22 µm centrifuge tube filter (Thermo Fisher Scientific, Waltham, MA, USA) and quantified using a BCA Protein Assay Kit (Beyotime, Shanghai, China). In total, 60 µg of total proteins were separated by 10% SurePAGE gel (GenScript, Nanjing, China) and transferred to 0.45 µm polyvinylidene difluoride (PVDF) membranes (Millipore, Billerica, MA, USA) using eBlot™ L1 (GenScript, Nanjing, China), followed blocked with TBS with 5% skim milk (BD, Franklin Lakes, NJ, USA)). Then membranes were respectively incubated with Bhmt antibodies (Proteintech, Rosemont, IL, USA), Rad23b antibodies (Abclonal, Wuhan, China), Cul4b antibodies (Abclonal, Wuhan, China), Rad23a antibodies (Abclonal, Wuhan, China), Rpa1 antibodies (Abclonal, Wuhan, China), Rpa3 antibodies (Abclonal, Wuhan, China), and beta Actin antibodies (Abcam, Cambridge, MA, USA) at 4 °C overnight and incubated with an anti-mouse HRP secondary antibody (Beyotime, Shanghai, China) and an anti-rabbit HRP secondary antibody (Cell Signaling Technology, Danvers, MA, USA) for 1 h at room temperature. The membranes were visualized using an ECL Substrate Kit (Millipore, Billerica, MA, USA).

### 4.7. Statistical Analysis

Statistical analysis was performed using GraphPad Prism 9.0.0. Results are shown as mean ± standard error of mean (SEM). Statistical significance was calculated with an unpaired *t*-test.

## Figures and Tables

**Table 1:** The sequences of PCR primers.

Primer Name	Forward Sequences (5′–3′)	Reverse Sequences (5′–3′)
*PCR-1*	GTGTGGCTGATGCGTCACAGAT	TGTCTCACGGTTGCCTGCTATT
*PCR-2*	CATTGCTCTTACCCAGTAGGGTCAG	CGACTTTCTCCTGGAGGAGTTCTGA

**Table 2:** The sequences of RT-qPCR primers.

Gene Name	Forward Sequences (5′-3′)	Reverse Sequences (5′-3′)
*RAD52*	GGTCCAGAGTACATTAGCAGC	ATGGAGTGTGCCCAGCCATT
*mt-Nd5*	GCTTATCCTCACCTCAGCCA	TGCTTGTAGGGCTGCAGTAT
*mt-Nd2*	CACGATCAACTGAAGCAGCA	TTGAGTACGATGGCCAGGAG
*mt-Nd1*	AGGATGAGCCTCAAACTCCA	GGTCAGGCTGGCAGAAGTAA
*mt-Cytb*	TTCATGTCGGACGAGGCTTA	TCCTCATGGAAGGACGTAGC
*mt-Co1*	CTGACTTGCAACCCTACACG	AGCAAACACTGCTCCCATTG
*Ercc2*	TCGCAAGGCTGTTGTGGTCTTC	CTTGATCCTGAGCACGGTCTTCTG
*Rad23b*	ACTTCCACTCCTGCCTCCACTG	GGTGTCTGGGCTGGCTTTTCTG
*Ddb1*	AGAGAAGGAGGAGCAGATGGATGG	GTTAGGCACTCAGCAATGGAGGTC
*Cul4b*	AACAGCAGCAGTAGCAGCAGTAAC	CAAGGCAGAAGGACGAGGTTGAAG
*Cul4a*	GCGGCTGCCTGACAACTACAC	CTGATGGATGTGCTGCTCTGGATG
*Rad23a*	AGGTTGGTGCCATAGGTGAGGAG	GCCCAGTGCCTTCAGCCTTTC
*Rpa3*	AGTATATCGACCGGCCCGTGTG	ATTTCCTCGTCAAGTGGCTCCATC
*Rpa1*	TGATGGGTTGAACACGCTTTCCTC	GATGAACTTGTGGACCTGGCAGAC
*Bdh2*	AGGCGGCTGTGATCGGTCTC	GTGCCTCTTTGGGATTGTCTCTGG
*Bhmt1b*	AAGCATCTGGTAAGCCCGTAGC	GCAGCCTCCAAACCCTCCTTC
*mt-Nd4*	GGGAACCAAACTGAACGCCTAAAC	GGCAATTAGCAGTGGAATAGAACCG
*Ppp1ccb*	TCAGGTGGTTGAAGATGGCTATGAG	GACAGGTCTCGTGGCGTTGG
*Ctnna2*	AACCTGATGAATGCTGTTGTCCTC	GCTGCTGTTCCATAGACCTTCTG
*Tulp3*	ACCTGATCGAGCTGCACAACAAG	ACGGATGCCTGGGTGACTCTG
*Golim4*	GGTGGTCTTCGGCTTCCTCTATG	CCTGGTGCTGCTGGTACTTGAG
*Retn*	ACTTCAACTCCCTGTTTCCAAATGC	GGCTGCTGTCCAGTCTATCCTTG
*β-actin*	GTGACGTTGACATCCGTAAAGA	GCCGGACTCATCGTACTCC

## Data Availability

Raw transcriptomic data could be downloaded from the China National Center for Bioinformation, National Genomics Data Center (accession: CRA021410). Raw proteomic data could be downloaded from OMIX (accession: OMIX008327).
